# Clinical outcome and MRI appearance in a group of chronic low back pain patients more than 10 years after discography evaluation and consideration for surgery

**DOI:** 10.1186/s12891-023-06242-y

**Published:** 2023-02-22

**Authors:** Hanna Hebelka, Veronica Gunterberg, Kerstin Lagerstrand, Helena Brisby

**Affiliations:** 1 Department of Radiology, Sahlgrenska University Hospital, Gothenburg, Sweden; 2 Institute of Clinical Sciences, Sahlgrenska Academy, University of Gothenburg, Gothenburg, Sweden; 3 Department of Orthopedics, Sahlgrenska University Hospital, Gothenburg, Sweden; 4 Department of Medical Physics and Biomedical Engineering, Sahlgrenska University Hospital, Gothenburg, Sweden

**Keywords:** Discography, Annular injury, Degeneration, Lumbar fusion, Patient reported outcome measure, MRI

## Abstract

**Background:**

It is an ongoing debate whether fusion surgery is superior to non-operative treatment for non-specific low back pain (LBP) in terms of patient outcome. Further, the evidence for how signs of intervertebral disc (IVD) degeneration on magnetic resonance imaging (MRI) correlate with patient outcome is insufficient. Longitudinal studies of low back pain (LBP) patients are thus of interest for increased knowledge. The aim of this study was to investigate long-term MRI appearance in LBP patients 11–14 years after discography.

**Methods:**

In 2021, 30 LBP patients who had same-day discography and MRI in 2007–2010 were asked to undergo MRI (Th12/L1–L5/S1), complete visual analog scale (VAS), Oswestry Disability Index (ODI) and EuroQol-5 Dimension (EQ5D) questionnaires. Patients who had fusion surgery before the follow-up were compared with those without such surgery. MRIs were evaluated on Pfirrmann grade, endplate classification score (EPS), and High Intensity Zones (HIZ). For each disk it was noted if injected at baseline or not.

**Results:**

Of 17 participants (6 male;mean age 58.5 years, range 49–72), 10 (27 disks) had undergone fusion surgery before the follow-up. No differences in VAS, ODI, or EQ5D scores were found between patients with and without surgery (mean 51/32/0.54 vs. 50/37/0.40, respectively; 0.77 > *p* < 0.65). Other than more segments with EPS ≥ 4 in the surgery group (*p* < 0.05), no between-group differences were found in longitudinal change in MRI parameters. Of 75 non-fused disks, 30 were injected at baseline. Differences were found between injected and non-injected disks at both baseline and follow-up for Pfirrmann grade and HIZ, and at follow-up for EPS (0.04 > *p* < 0.001), but none for progression over time (0.09 > *p* < 0.82).

**Conclusions:**

Other than more endplate changes in the surgery group, no differences in longitudinal change of MRI parameters were established between LBP patients treated with or without fusion surgery in the studied cohort. The study also highlights the limited progress of degenerative changes, which may be seen over a decade, despite needle puncture and chronic LBP.

## Background

Low back pain (LBP) is a major cause of disability worldwide [1, 2], affecting up to 80% of both men and women during their lifetime. Treatment strategies vary, and the role of surgery is under constant scrutiny [3, 4]. Whether fusion surgery is superior overall to non-operative treatment for non-specific LBP in terms of patient outcome remains a subject of ongoing debate [3, 4], as does whether strengthened diagnostic criteria could be used to select sub-groups of LBP patients with greater chances of success with a specific treatment [5–8]. Despite these uncertainties, annual fusion surgeries have increased by up to 500% since the early1990s [9–11]. LBP is known to be associated with intervertebral disk (IVD) degeneration, however the exact interplay between degenerative findings in the spine and LBP is still unclear [12]. Furthermore, the development of degenerative changes over time, both for individuals and for specific spinal motion segments, is difficult to predict. Longitudinal studies of LBP patients are of interest to increase knowledge in this area.

Annular injury of the IVD has been demonstrated, in both experimental animal studies and human studies, to induce and accelerate IVD degeneration [13–16]. One way to study annular injuries caused by needle puncture in humans is to study people whose IVDs were punctured for a specific reason such as discography [16]. Provocative discography, an imaging-guided procedure in which a contrast agent is injected into the nucleus pulposus of the IVD, to evaluate pain response and obtain detailed IVD characteristics, was previously believed to be a useful method for patient selection. However studies have not confirmed its usefulness [17, 18] and it has been demonstrated to accelerate degenerative changes in punctured IVDs [16], why discography is no longer used at our center. However, although discography is now rarely used, long-term follow-up of individuals with previous discography can provide an in vivo human model for annular injuries and might thus provide deeper insight into the association between annular injury and the degenerative process [16]. This is important not only to increase knowledge of the pathophysiology of degeneration, but also since novel therapeutic interventions may involve disk puncture, such as cell therapy with injection of platelet-rich plasma and growth factors [5–8].

The aim of this study was to investigate long-term magnetic resonance imaging (MRI) appearance of the lumbar spine in a group of patients with LBP 11–14 years after discography.

## Methods

Thirty LBP patients, prospectively enrolled from 2007 to 2010 in a comparative discography/MRI study (1.5 T, sagittal T2/T1-weighted) [19] were considered for participation in this long-term follow-up. Exclusion criteria for the current study was inability to undergo MRI. The reason for discography at inclusion was history of at least 6 months’ non-specific LBP, severe enough to consider surgery. All referred patients had failed conservative therapy. None of the patients had any radiculopathy. Pressure-controlled discography was performed using a manual manometer (Stryker Discmonitor, Kalamazoo, Michigan) with approximately 0.2 mL injected contrast (Omnipaque 180 mg/mL, GE Healthcare, Oslo, Norway) at each twist. Details of the discography procedure have previously been described [19].

**Fig. 1:**
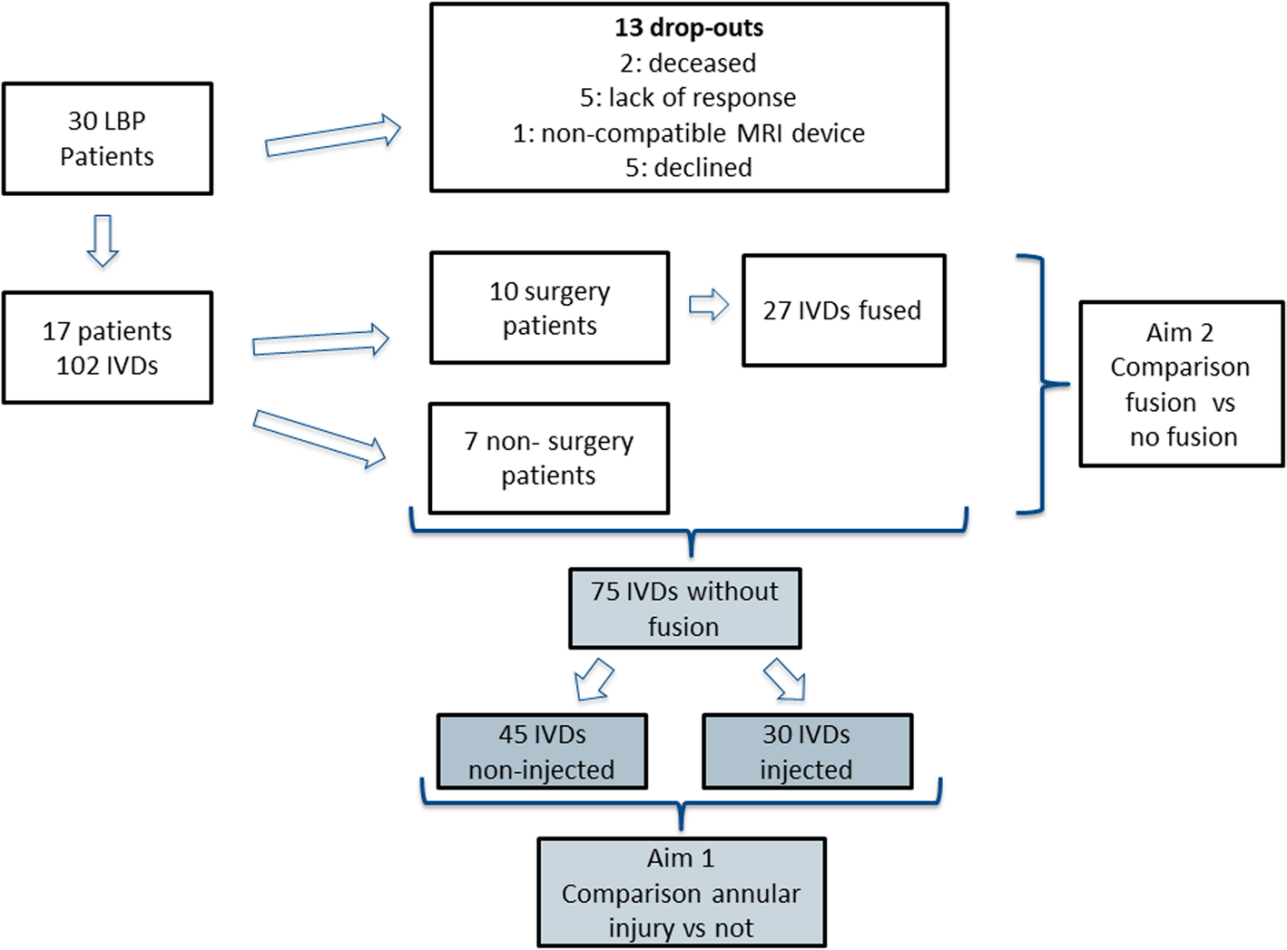
Schematic patient flow chart

In 2021, those agreeing to participate underwent an MRI of the lumbar spine and filled in validated pain, function, and quality of life questionnaires (visual analog scale [VAS], Oswestry Low Back Pain Disability Index [ODI], and EuroQol-5 Dimension [EQ5D]; Fig. 1). Baseline and follow-up MRIs were performed on the same scanner (Siemens Magnetom Symphony Maestro Class, Erlangen, Germany) with at minimum T1-weighted sagittal (TR 541 ms/TE 1 ms) and T2-weighted sagittal (TR 4000 ms/TE 124 ms) images (slth4mm/FoV300mm) obtained. The MRIs were evaluated according to Pfirrmann classification [20], Endplate Classification Score (EPS) [21], and HIZ [22] for six IVDs per individual at both baseline and follow-up (Th12/L1–L5/S1). The MRI grading was performed by a senior radiologist (> 15 years’ experience with spinal MRI) blinded to whether or not each IVD had been injected with contrast during the discography procedure at baseline. After a month the same radiologist, blinded to the previous evaluation, again evaluated the MRIs to assess intra-rater agreement measures.

To investigate long-term patient-reported outcome (PROM) between the groups of patients with and without fusion surgery, each IVD segment (Th12/L1–L5/S1) was classified by whether or not any type of fusion surgery had been performed between baseline and follow-up. IVDs were dichotomized according to Pfirrmann ≥3 or < 3, with HIZ or not, and segments with EPS ≥4 or < 4 per individual. Fused segments were allocated to the high score Pfirrmann and EPS groups. These data were compared between the group of patients who had surgery (any number of segments) during the follow-up period and those who had not. The flow chart is displayed in Fig. 1.

To investigate longitudinal MRI appearance between IVDs with and without annular puncture, segments fused during the follow-up period were excluded from the analysis and the non-fused injected IVDs were compared with non-injected IVDs (Fig. 1). At baseline, discography of 2 to 4 IVDs had been performed in each individual.

The study was approved by the National Ethical Review Board (Dnr 366–07 and Dnr 2020–03511) and all procedures were performed in accordance with the 1964 Helsinki declaration and its later amendments. Informed consent was obtained from all subjects.

### Statistics

Descriptive statistics were used, with n (%) for categorical variables and mean (SD) for continuous variables. For comparisons of parameters over time, the Wilcoxon Signed Rank test was used for continuous variables and Sign test for categorical variables. For between-group comparisons, Mann-Whitney U-test was used for continuous variables, Fisher’s Exact test (lowest 1-sided *p*-value multiplied by 2) for dichotomous variables, and Mantel-Haenszel Chi Square test for ordered categorical variables. The data were analyzed using version 9.4 of the SAS System. A *p*-value of < 0.05 was considered significant.

The intraclass correlation coefficients (ICC) with 95% confidence intervals, model 2 with absolute agreement, was used for Pfirrmann grading and EPS, and Cronbach’s alfa coefficient for the dichotomized HIZ [23].

## Results

Seventeen (6 male/mean 58.5 years; range 49–72) of thirty invited patients agreed to participate in this 11- to 14-year follow-up. Reasons for not participating were; 2 deceased, 5 unreachable, 1 with a non-MRI compatible device, and 5 who declined participation (Fig. 1).

Between baseline and follow-up, ten patients (27 IVDs) had undergone fusion surgery (Fig. 1). Eight patients had undergone posterolateral instrumented fusion [1 level (*n* = 4), 2 levels (*n* = 2), 3 levels (*n* = 1), 5 levels (*n* = 1)]. One patient initially obtained a disc prosthesis and later a posterolateral instrumented fusion Th10-L5 was performed. One patient was fused over three levels with combined anterior and posterolateral approach.

**Fig. 2:**
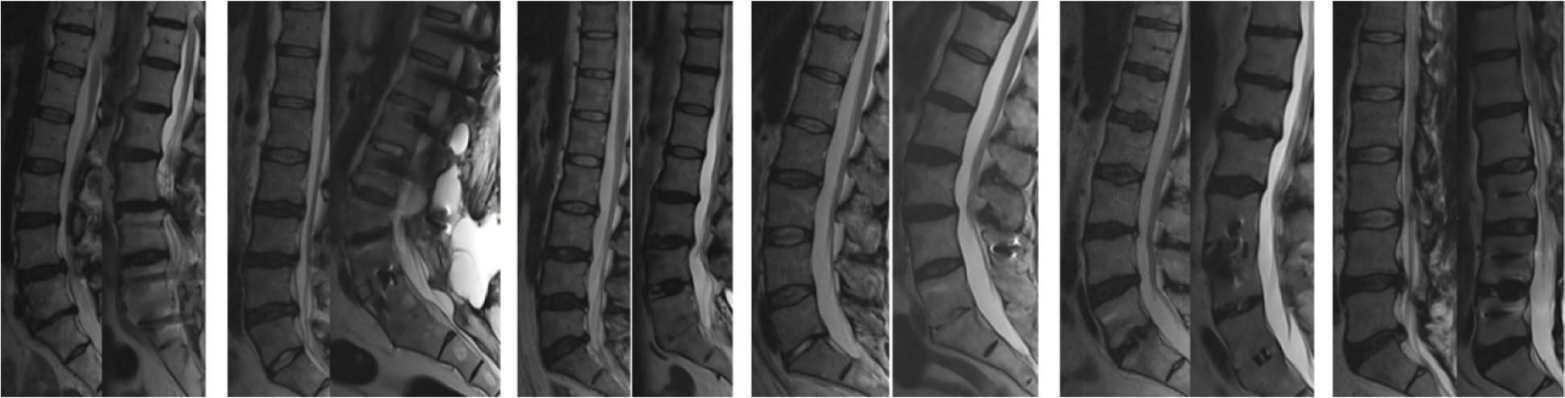
Examples of Magnetic resonance imaging appearance in the surgery group. Example of 6 pairs of sagittal MRI at baseline (left) and 11- to 14-year follow-up (right) for some of the individuals who had undergone fusion surgery during the follow-up period

No significant differences were found in back pain score (VAS) at the follow-up between fusion patients and patients not surgically treated (*p* = 0.72), with both groups having a mean VAS of about 50, nor were there any between-group differences in ODI or EQ5D scores (Table 1). No significant differences for MRI parameters at baseline or at follow-up was found, other than significantly more HIZ IVDs at follow-up in the non-surgery group (*p* < 0.05). Comparing the groups regarding longitudinal changes of MRI parameters, no differences were found, other than more segments with EPS ≥ 4 in the surgery group (*p* < 0.05) (Table 2; Fig. 2).

**Table 1:** Patient-reported outcome measures for the group of patients with and without fusion surgery at follow-up

Follow-up evaluation	No surgery (***n*** = 7)	Surgery (***n*** = 10)	***P***-value
**VAS**	51.3 (20.7)	50.4 (28.8)	0.72
**ODI**	32.0 (19.1)	36.6 (22.5)	0.77
**EQ5D**	0.54 (0.39)	0.40 (0.45)	0.65

**Table 2:**
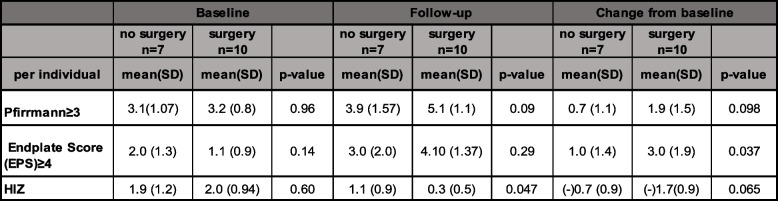
Magnetic resonance imaging parameters at baseline and follow-up for patients with and without fusion surgery during the follow-up period

**Fig. 3:**
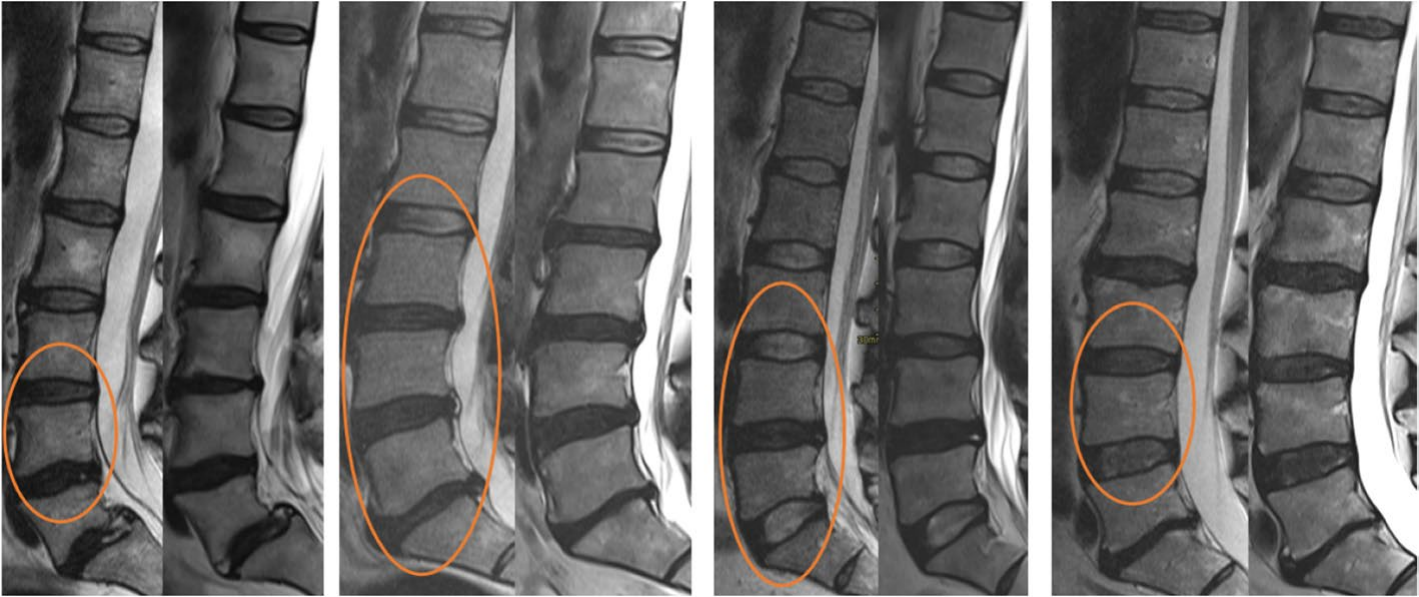
Examples of MRI appearance in the non-surgery group. Example of 4 pairs of sagittal MRI at baseline (left) and 11- to 14-year follow-up (right) for some of the individuals who had not undergone fusion surgery during the follow-up period. Injected intervertebral disks are highlighted with a circle

Of the 75 IVDs not included in fused segments, 30 had been injected during discography at baseline (Fig. 1). Significant differences in MRI parameters were found between injected and non-injected IVDs at both baseline and follow-up for distribution of Pfirrmann grade and HIZ, and also for EPS at follow-up, with higher degeneration grades and EPS in injected IVDs (Table 3). However, in terms of morphological changes over time, no significant differences were detected between injected and non-injected IVDs (Table 3; Fig. 3).

**Table 3:**
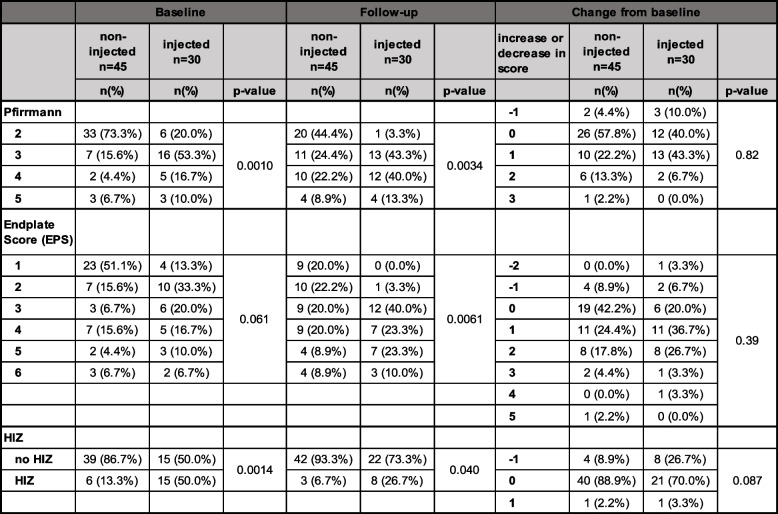
Magnetic resonance imaging parameters at baseline and follow-up for injected and non-injected IVDs

For Pfirrmann grading and EPS, the ICC for intra-observer agreement was high (0.91 [95% confidence interval (CI 0.85–0.94) and 0.91[95% CI 0.84–0.95]). Also the agreement in assessment of HIZ was high with Chronbach alfa 0.76.

## Discussion

In this group of patients with chronic LBP severe enough to be considered for fusion surgery at baseline, no differences in PROMs could be established at the 11- to 14-year follow-up between patients treated with or without fusion surgery. No significant differences in progression of degenerative changes were detected between discography-injected and non-injected IVDs after more than 10 years, although the small cohort in this study is an inherent limitation. This long-term follow-up could therefore not confirm previous findings that needle-puncture of the disk (annular injury) accelerates the degenerative progression of the IVD [16]. This reflects the complexity of the relation between LBP and disk degeneration, suggesting that neither long-term PROMs nor MRI progression of degeneration can be anticipated from the appearance at baseline MRI.

Based on the small number of patients included, it is of course difficult to make any firm conclusions whether differences between patient groups, PROMs, or injected/non-injected disks exists since one might argue that this study is highly under-powered and there could be many contributing factors that influence prime variables during the ten-year follow-up. However, it is likely that some differences would be seen after such a long follow-up period if clinically relevant differences do exist. The current findings are in line with available evidence, and do not show a convincing benefit of spine fusion over non-operative alternatives for back pain associated with disk degeneration [4, 10].

Because, with the exception of injected control IVDs, discographies had been performed on IVDs suspected to be the pain source of LBP and thus more degenerated in general, we expected that injected IVDs would have higher baseline Pfirrmann grades, higher EPS, and more HIZs than non-injected. The majority of the observed IVDs, both injected and non-injected, showed moderate progression of degeneration, which was also expected, considering the long follow-up period of 11 to14 years, however without significant longitudinal changes between the injected and non-injected IVDs.

Studies evaluating long-term clinical and MRI follow-up of patients exposed to annular injury and injection are rare [16, 24]. To our knowledge only the study by Carragee et al. performed such a systematic study with controls [16]. In that study, individuals who had undergone discography and controls not exposed to discography were followed-up after 7 to 10 years. The researchers found faster degeneration in punctured IVDs (L3/4–L5/S1) than in corresponding non-injected IVDs at these levels in the control cohort, but no difference between the non-punctured IVDs (L1/2–L2/3) in the discography group and the corresponding IVDs in the control group. Even if these individuals were matched for age, sex, and previous spinal symptoms, it remains possible that between-individual factors might have influenced those results. It is known that IVDs of the lower lumbar spine are more prone to degeneration, which might at least partly explain why between-group differences were detected in the lower lumbar spine as opposed to at levels in the upper lumbar spine, e.g.L1/L2–L2/3.

Although the current study is limited by its small sample size, with a high risk of type II error, one of its strengths is the elimination of any between-individual bias in the progression of degeneration since both injected and non-injected IVDs existed within each individual. On the other hand, the current study may be limited by this method, since injected levels are most often situated in the lower lumbar spine and non-injected IVDs in the upper. Another important methodological factor that likely contributed to the discrepancy in results between the studies is differences in the cohorts studied. The cohorts in the study by Carragee et al. had mild LBP and were recruited from a cohort with a history of previous cervical/lumbar disk herniation, while all patients in the current study had LBP severe enough to be considered for fusion surgery. In the study by Carragee et al., new herniations constituted a majority of the new events in the discography cohort. Since a heritable predisposition for disk herniation and degenerative disk disease has been reported [25, 26], hereditary factors making the nucleus pulposus and/or annulus fibrosus more prone to new herniations when injured (injected) might have influenced the results.

Annular injuries of substantial size are unquestionably associated with degeneration [15], and we do not advocate the use of discography. However, it is interesting to note the surprisingly limited progress of degenerative changes seen after over a decade in many of the individuals examined, in spite of needle punctures, contrast injection, and symptoms of LBP (Fig. 3). In accordance with these current results, Carragee et al. also highlighted that over 50% of injected IVDs with Pfirrmann grade 1–2 at baseline remained stable over a 10-year period [16]. Wai et al. also reported in their 20-year MRI follow-up after anterior lumbar fusion that in 39 patients with normal preoperative discograms at adjacent levels, only 14% of these non-fused (injected) segments displayed signs of advanced degeneration [24]. They had no control cohort, but concluded that their prevalence of degenerative findings was similar to results in asymptomatic controls in the literature. This demonstrates the complex interplay between annular injury and disk degeneration and implies that this process apparently is multifactorial and difficult to predict.

## Limitations

As a small cohort study, this project had inherent limitations, especially as a relatively large proportion of the initial cohort was not available for the long-term follow-up. There could be many contributing factors that influence prime variables during the ten-year follow-up, however the small sample size did not allow multivariate analysis. Despite this, it is interesting to see how surprisingly little progression of degenerative changes that is seen in some individuals, despite punctured and contrast-injected IVDs in patients with LBP pain of degree considering surgery. Selection bias for surgery/non-surgery in a pre-selected cohort study may also have affected the clinical results. Since baseline PROM data were not available, between-group PROM differences at baseline and over time could not be investigated. Despite these limitations, however, the study’s strengths are that follow-up MRI as well as clinical information was obtained for both pain and function 11 to 14 years after disk puncture in a group of patients with LBP severe enough to be considered for fusion surgery.

## Conclusions

No between-group differences were established in longitudinal changes of MRI parameters in LBP patients treated or not treated with fusion surgery other than more endplate changes in the surgery group. The study also highlights the limited progress of individual degenerative changes, which may be seen after over a decade in spite of needle puncture and chronic LBP. These findings demonstrate the high complexity of annular injury and disk degeneration progression and suggest that other factors may be of greater importance than limited annular injuries in the development of disk degeneration.

## Data Availability

The datasets used and/or analyzed during the current study are available from the corresponding author on reasonable request.

## Abbreviations

EQ5D: EuroQol-5 Dimension
HIZ: High Intensity Zone
IVD: Intervertebral disc
LBP: Low back pain
MRI: Magnetic resonance imaging
ODI: Oswestry Disability Index
PROM: Patient Reported Outcome Measure
VAS: Visual analog scale

## References

[1] Manchikanti L, Benyamin RM, Singh V (2013). An update of the systematic appraisal of the accuracy and utility of lumbar discography in chronic low back pain. Pain Physician.

[2] Hoy D, March L, Brooks P (2014). The global burden of low back pain: estimates from the global burden of disease 2010 study. Ann Rheum Dis.

[3] Zhao L, Manchikanti L, Kaye AD, Abd-Elsayed A (2019). Treatment of discogenic low back pain: current treatment strategies and future options—a literature review. Curr Pain Headache Rep.

[4] Harris IA, Traeger A, Stanford R, Maher CG, Buchbinder R (2018). Lumbar spine fusion: what is the evidence?. Internal Med J.

[5] Pettine KA, Suzuki RK, Sand TT, Murphy MB (2017). Autologous bone marrow concentrate intradiscal injection for the treatment of degenerative disc disease with three-year follow-up. Int Orthop.

[6] Akeda K, Ohishi K, Masuda K (2017). Intradiscal injection of autologous platelet-rich plasma releasate to treat discogenic low back pain: a preliminary clinical trial. Asian Spine J.

[7] Muthu S, Jeyaraman M, Chellamuthu G, Jeyaraman N, Jain R, Khanna M (2021). Does the intradiscal injection of platelet rich plasma have any beneficial role in the management of lumbar disc disease?. Global Spine J.

[8] Hirase T, Jack Ii RA, Sochacki KR, Harris JD, Weiner BK (2020). Systemic review: is an intradiscal injection of platelet-rich plasma for lumbar disc degeneration effective?. Cureus..

[9] HCUPnet. Agency for Healthcare Research and Quality. Available at: http://hcupnet.ahrq.gov/HCUPnet.jsp. Accessed 30 Oct 2014.

[10] Deyo RA (2015). Fusion surgery for lumbar degenerative disc disease: still more questions than answers. Spine J.

[11] Rajaee SS, Bae HW, Kanim LE, Delamarter RB (2012). Spinal fusion in the United States: analysis of trends from 1998 to 2008. Spine..

[12] Urban JP, Fairbank JC (2020). Current perspectives on the role of biomechanical loading and genetics in development of disc degeneration and low back pain; a narrative review. J Biomech.

[13] Newton MD, Marek AA, Planalp M, Park DK, Baker KC, Maerz T (2020). Longitudinal characterization of intervertebral disc remodeling following acute annular injury in a rat model of degenerative disc disease. Connect Tissue Res.

[14] Hadjipavlou A, Simmons J, Pope M, Necessary J, Goel V (1999). Pathomechanics and clinical relevance of disc degeneration and annular tear: a point-of-view review. Am J Orthop (Belle Mead, NJ).

[15] Osti O, Vernon-Roberts B, Moore R, Fraser R (1992). Annular tears and disc degeneration in the lumbar spine. A post-mortem study of 135 discs. J Bone Joint Surg Br.

[16] Carragee EJ, Don AS, Hurwitz EL, Cuellar JM, Carrino J, Herzog R (2009). 2009 ISSLS prize winner: does discography cause accelerated progression of degeneration changes in the lumbar disc: a ten-year matched cohort study. Spine..

[17] Hebelka H, Nilsson A, Hansson T (2013). Pressure increase in adjacent discs during clinical discography questions the methods validity. Spine (Phila Pa 1976).

[18] Hebelka H, Nilsson A, Ekstrom L, Hansson T (2013). In vivo discography in degenerate porcine spines revealed pressure transfer to adjacent discs. Spine (Phila Pa 1976).

[19] Hebelka H, Hansson T (2013). HIZ's relation to axial load and low back pain: investigated with axial loaded MRI and pressure controlled discography. Eur Spine J.

[20] Pfirrmann CW, Metzdorf A, Zanetti M, Hodler J, Boos N (2001). Magnetic resonance classification of lumbar intervertebral disc degeneration. Spine (Phila Pa 1976).

[21] Rajasekaran S, Babu JN, Arun R, Armstrong BRW, Shetty AP, Murugan S (2004). ISSLS prize winner: a study of diffusion in human lumbar discs: a serial magnetic resonance imaging study documenting the influence of the endplate on diffusion in normal and degenerate discs. Spine..

[22] Peng B, Hou S, Wu W, Zhang C, Yang Y (2006). The pathogenesis and clinical significance of a high-intensity zone (HIZ) of lumbar intervertebral disc on MR imaging in the patient with discogenic low back pain. Eur Spine J.

[23] Cicchetti DV (1976). Assessing inter-rater reliability for rating scales: resolving some basic issues. Br J Psychiatry.

[24] Wai EK, Santos ER, Morcom RA, Fraser RD (2006). Magnetic resonance imaging 20 years after anterior lumbar interbody fusion. Spine..

[25] Patel AA, Spiker WR, Daubs M, Brodke D, Cannon-Albright LA (2011). Evidence for an inherited predisposition to lumbar disc disease. J Bone Joint Surg Am.

[26] Theodore N, Ahmed AK, Fulton T (2019). Genetic predisposition to symptomatic lumbar disk herniation in pediatric and young adult patients. Spine..

